# Occupational disparities in common cancer screening participation among workers: a nationwide cross-sectional study in Japan

**DOI:** 10.1093/joccuh/uiaf046

**Published:** 2025-09-08

**Authors:** Kazuhiko Watanabe, Takahiro Tabuchi, Masayoshi Zaitsu

**Affiliations:** Center for Research of the Aging Workforce, University of Occupational and Environmental Health, Japan, Kitakyushu, Fukuoka, Japan; Division of Epidemiology, School of Public Health, Graduate School of Medicine, Tohoku University, Sendai, Miyagi, Japan; Center for Research of the Aging Workforce, University of Occupational and Environmental Health, Japan, Kitakyushu, Fukuoka, Japan

**Keywords:** cancer screening, occupational class, disparity, manual worker, prognosis

## Abstract

**Objectives:**

Cancer screening is crucial for early detection and improved health outcomes. Limited evidence exists on the association between occupational class and cancer screening participation in Japan. Therefore, we aimed to examine screening participation rates and disparities among active workers across different occupational classes.

**Methods:**

This cross-sectional study analyzed data from a nationwide web-based survey conducted in Japan (September to November, 2023). Eligible participants included current workers aged 40-64 years for colorectal, lung, and stomach cancer screenings (*n* = 7038); workers aged 40-64 years for breast cancer screening (*n* = 2929); and workers aged 30-64 years for cervical cancer screening (*n* = 4252). Cancer screening participation rates across occupational classes (upper nonmanual, lower nonmanual, and manual workers) were compared using the chi-square test. Poisson regression with robust variance was used to estimate prevalence ratios (PRs) and 95% CIs for nonparticipation, adjusted for sex, age, educational attainment, household income, and workplace scale. Upper nonmanual workers served as the reference group.

**Results:**

Manual workers consistently had lower cancer screening participation rates. Compared with upper nonmanual workers, manual workers exhibited significantly higher PRs for nonparticipation in colorectal (PR = 1.12; 95% CI, 1.04-1.22), lung (PR = 1.22; 95% CI, 1.12-1.34), stomach (PR = 1.14; 95% CI, 1.05-1.23), and cervical cancer screenings (PR = 1.16; 95% CI, 1.02-1.33). The disparities were particularly pronounced among male workers.

**Conclusions:**

Manual workers had lower cancer screening participation rates, particularly for colorectal, lung, stomach, and cervical cancer. Targeted interventions are needed to improve screening, particularly among manual workers, and reduce occupational disparities in cancer prevention and outcomes.

## Teaser

Key points

- **What is already known on this topic:** Cancer prognostic disparities are linked to occupational class and can result in poor outcomes among manual workers, with mortality risk partly attributed to advanced cancer stages at diagnosis.
- **What this study adds:** Manual occupation was associated with nonparticipation in colorectal, lung, stomach, and cervical cancer screening.
- **How this study might affect research, practice, or policy:** This study highlights the need to promote cancer screening participation among manual workers to reduce occupational disparities in cancer prognoses.

## 1. Background

Cancer is the leading cause of death worldwide.^1^ In 2020, an estimated 19.3 million new cases were diagnosed globally, with approximately 10 million cancer-related deaths.^2^ The burden of cancer, measured in disability-adjusted life-years, continues to rise due to population growth and aging.^2^ As a major noncommunicable disease, cancer not only reduces healthy life expectancy but also causes significant economic losses.^3^ In Japan, the lifetime cancer incidence is 62.1% in men and 48.9% in women, with approximately 950 000 new cases diagnosed in 2020.^4^ Cancer has remained the leading cause of death for over 40 years, with mortality rates continuing to rise.^5^ In 2023, an estimated 380 000 cancer-related deaths accounted for 24.3% of all deaths.^4^ Given this substantial burden, effective strategies for cancer diagnosis, treatment, and prevention are crucial.

Prior research has highlighted disparities in cancer prognosis based on occupational class.^6-8^ Individuals in manual occupations, such as manufacturing, construction, and transportation, have lower 5-year cancer survival rates compared with those in upper nonmanual positions (professional and managerial roles).^8^ One key factor contributing to this disparity is late-stage diagnosis, which accounts for approximately one-third of the increased mortality risk.^8^ Since early detection through screening improves cancer outcomes, understanding differences in screening participation across occupational classes is essential.

In Japan, nationwide cancer screening programs are recommended for colorectal, lung, stomach, breast, and cervical cancers based on specific age criteria.^9^ However, differences in cancer screening participation by occupational class within this system remain poorly understood.

In this study, we aimed to examine cancer screening participation rates for common cancer sites across different occupational classes using a nationwide dataset. Additionally, we investigated the association between occupational class and nonparticipation in cancer screening.

## 2. Methods

### 2.1. Study setting and participants

This nationwide cross-sectional study used data from the Japan COVID-19 Society Internet Survey (JACSIS) (https://jacsis-study.jp/), conducted in September and November 2023. The JACSIS dataset includes approximately 2.3 million panelists from all 47 prefectures in Japan. Details regarding JACSIS, including quality controls, national representativeness, and panelist policies, have been published previously.^10^^,^^11^

Initially, 28 481 participants aged 16-83 years were included in this study. Of these, 9163 individuals who were not actively employed (eg, homemakers, students, and unemployed individuals), as well as those engaged in security jobs (*n* = 256) or other occupations (*n* = 3637) were excluded.^12^ The final study population was defined based on the recommended screening age for each cancer site.^9^ Specifically, workers aged 40-64 years were included for colorectal, lung, stomach, and breast cancer screening, whereas those aged 30-64 years were included for cervical cancer screening. We excluded individuals aged ≤39 or ≥65 years from the colorectal, lung, and stomach cancer analyses (*n* = 8387). For the breast cancer screening analysis, we further excluded those with missing screening information (*n* = 4109). Similarly, for the cervical cancer screening analysis, we excluded individuals aged ≤29 or ≥65 years (*n* = 5377), along with those with missing screening information (*n* = 5796). The final analytical sample, comprising only participants with complete data for all variables, included 7038 workers for colorectal, lung, and stomach cancer screenings (mean age ± SD, 51.1 ± 7.0 years), 2929 workers for breast cancer screening (50.9 ± 6.9 years), and 4252 workers for cervical cancer screening (45.8 ± 9.7 years).

Informed consent was obtained electronically from all participants before completing the internet-based questionnaire. This study was conducted in accordance with the principles of the Declaration of Helsinki (1964) and was approved by the Ethics Committee of the University of Occupational and Environmental Health, Japan (No. R4-054).

### 2.2. Main outcome: nonparticipation in cancer screening

The main outcome was nonparticipation in cancer screening. We calculated the participation rates of cancer screening at each recommended site, where screening is advised either annually or biennially in Japan.^9^ Cancer screening participation was assessed based on responses to the question: “Did you undergo cancer screening in the past 1 or 2 years?”

The response options were:

1. Underwent screening; no abnormalities detected.
2. Underwent screening; abnormalities were detected.
3. Underwent screening; results unknown.
4. Did not undergo screening for reasons unrelated to COVID-19, but intends to do so in the future.
5. Did not undergo screening for COVID-19–related reasons, but intends to do so in the future.
6. Did not undergo screening for reasons unrelated to COVID-19 and does not intend to do so in the future.
7. Did not undergo screening for COVID-19–related reasons and does not intend to do so in the future.

Participants reported screening attendance within the past year for colorectal, lung, and stomach cancer and within the past 2 years for breast and cervical cancer. Those who selected options 1, 2, or 3 were classified as “participation,” whereas those who selected options 4, 5, 6, or 7 were classified as “nonparticipation.”

The screening methods for each cancer site were as follows:

1. Colorectal cancer: fecal occult blood test (stool test).
2. Lung cancer: chest X-ray or sputum cytology.
3. Stomach cancer: barium X-ray or endoscopy (gastroscopy and fiberscopy).
4. Breast cancer: mammography or ultrasound.
5. Cervical cancer: cervical cytology.

### 2.3. Occupational class and other covariates

Using current occupational data based on the Erikson-Goldthorpe-Portocarero scheme^12^ and the Japan Standard Occupational Classification,^13^ we categorized participants into 3 occupational classes: upper nonmanual (professional and managerial workers), lower nonmanual (clerical, sales, and service occupations), and manual workers (occupations in manufacturing, transport, and machine operation, construction, and mining, as well as carrying, cleaning, and packaging roles).

Potential confounders included sex, age, educational attainment (high school or lower), and household income per capita (<1.5 million JPY [Japanese yen], ≥1.5 million JPY, or unknown). Other covariates included workplace size (1-49, 50-999, ≥1000 employees, or unknown).^14^

### 2.4. Statistical analyses

Differences in cancer screening participation rates across occupational classes were assessed using chi-squared tests. Poisson regression with robust variance estimation was performed to calculate prevalence ratios (PRs) and 95% CIs for nonparticipation in cancer screening, with upper nonmanual workers serving as the reference group. Model 1 was adjusted for potential confounders, whereas Model 2 was adjusted for workplace size. For the sensitivity analysis, we applied the same methods to participants stratified by age: 40-49 years and 50-64 years for colorectal, lung, stomach, and breast cancer screening, and 30-49 years and 50-64 years for cervical cancer screening. Additionally, for the subgroup analysis, we conducted sex-stratified assessments for colorectal, lung, and stomach cancer screening. All statistical tests were 2-sided with an α level of .05. Analyses were conducted using STATA/SE 17.0 (StataCorp LLC, College Station, TX, USA).

## 3. Results

### 3.1. Participant characteristics

Among the 7038 workers included in the analysis, 2929 (41.6%) participants in the colorectal, lung, and stomach cancer screenings were women (Table 1). All participants in the breast and cervical cancer screening analyses were women (Tables 2 and 3). Approximately half of the manual workers across all cancer sites had an educational attainment of high school or lower (Tables 1, 2, and  3).

**Table 1:** Characteristics of study participants for colorectal, lung, and stomach cancer screening.

**Variable**	***n* (%) or mean [SD]**			
	**Total**	**Upper nonmanual** ^**a**^	**Lower nonmanual** ^**a**^	**Manual** ^**a**^
*n*	7038	1874	4093	1071
Colorectal cancer screening				
Participation	3648 (51.8)	**1039 (55.4)**	**2116 (51.7)**	**493 (46.0)**
Nonparticipation	3390 (48.2)	**835 (44.6)**	**1977 (48.3)**	**578 (54.0)**
Lung cancer screening				
Participation	4130 (58.7)	**1196 (63.8)**	**2388 (58.3)**	**546 (51.0)**
Nonparticipation	2908 (41.3)	**678 (36.2)**	**1705 (41.7)**	**525 (49.0)**
Stomach cancer screening				
Participation	3783 (53.8)	**1082 (57.7)**	**2196 (53.7)**	**505 (47.2)**
Nonparticipation	3255 (46.2)	**792 (42.3)**	**1897 (46.3)**	**566 (52.8)**
Female sex	2929 (41.6)	**587 (31.3)**	**2086 (51.0)**	**256 (23.9)**
Age	51.1 [7.0]	51.2 [7.3]	51.1 [7.0]	50.9 [6.7]
Educational attainment with high school or lower	1794 (25.5)	**181 (9.7)**	**1053 (25.7)**	**560 (52.3)**
Household income				
<1.5 million yen	227 (3.2)	**37 (2.0)**	**132 (3.2)**	**58 (5.4)** ^b^
≥1.5 million yen	5465 (77.6)	**1485 (79.2)**	**3171 (77.5)**	**809 (75.5)** ^b^
Unknown	1346 (19.1)	**352 (18.8)**	**790 (19.3)**	**204 (19.0)** ^b^
Workplace size^b^				
1-49	2297 (32.6)	**566 (30.2)**	**1378 (33.7)**	**353 (33.0)**
≥50 to <1000	2334 (33.2)	**657 (35.1)**	**1293 (31.6)**	**384 (35.9)**
≥1000	1904 (27.1)	**557 (29.7)**	**1133 (27.7)**	**214 (20.0)**
Unknown	503 (7.1)	**94 (5.0)**	**289 (7.1)**	**120 (11.2)**

a Boldface indicates *P* value <.05.

b Percentages do not total 100% due to rounding.

**Table 2:** Characteristics of female participants who responded to the breast cancer screening question.

**Variable**	***n* (%) or mean [SD]**			
	**Total**	**Upper nonmanual** ^**a**^	**Lower nonmanual** ^**a**^	**Manual** ^**a**^
*n*	2929	587	2086	256
Breast cancer screening				
Participation	1679 (57.3)	**338 (57.6)**	**1217 (58.3)**	**124 (48.4)**
Nonparticipation	1250 (42.7)	**249 (42.4)**	**869 (41.7)**	**132 (51.6)**
Female sex	2929 (100.0)	587 (100.0)	2086 (100.0)	256 (100.0)
Age	50.9 [6.9]	50.7 [7.2]	50.9 [6.9]	51.3 [6.6]
Educational attainment with high school or lower	819 (28.0)	**33 (5.6)**	**654 (31.4)**	**132 (51.6)**
Household income				
<1.5 million yen	119 (4.1)	**10 (1.7)**	**88 (4.2)**	**21 (8.2)**
≥1.5 million yen	2074 (70.8)	**426 (72.6)**	**1472 (70.6)**	**176 (68.8)**
Unknown	736 (25.1)	**151 (25.7)**	**526 (25.2)**	**59 (23.0)**
Workplace size^b^				
1-49	1049 (35.8)	**221 (37.6)**	**739 (35.4)**	**89 (34.8)**
≥50 to <1000	912 (31.1)	**205 (34.9)**	**625 (30.0)**	**82 (32.0)**
≥1000	633 (21.6)	**115 (19.6)**	**483 (23.2)**	**35 (13.7)**
Unknown	335 (11.4)	**46 (7.8)**	**239 (11.5)**	**50 (19.5)**

a Boldface indicates *P* value <.05.

b Percentages do not total 100% due to rounding.

**Table 3:** Characteristics of female participants who responded to the cervical cancer screening question.

**Variable**	***n* (%) or mean [SD]**			
	**Total**	**Upper nonmanual** ^**a**^	**Lower nonmanual** ^**a**^	**Manual** ^**a**^
*n*	4252	937	2975	340
Cervical cancer screening				
Participation	2349 (55.2)	**552 (58.9)**	**1639 (55.1)**	**158 (46.5)**
Nonparticipation	1903 (44.8)	**385 (41.1)**	**1336 (44.9)**	**182 (53.5)**
Female sex	4252 (100.0)	937 (100.0)	2975 (100.0)	340 (100.0)
Age	45.8 [9.7]	**44.5 [10.0]**	**46.1 [9.6]**	**47.3 [9.1]**
Educational attainment with high school or lower	1024 (24.1)	**44 (4.7)**	**804 (27.0)**	**176 (51.8)**
Household income				
<1.5 million yen	154 (3.6)^b^	**13 (1.4)**	**114 (3.8)**	**27 (7.9)**
≥1.5 million yen	3114 (73.2)^b^	**713 (76.1)**	**2163 (72.7)**	**238 (70.0)**
Unknown	984 (23.1)^b^	**211 (22.5)**	**698 (23.5)**	**75 (22.1)**
Workplace size^b^				
1–49	1415 (33.3)	**312 (33.3)**	**989 (33.2)**	**114 (33.5)**
≥50, <1000	1323 (31.1)	**319 (34.0)**	**896 (30.1)**	**108 (31.8)**
≥1000	987 (23.2)	**203 (21.7)**	**733 (24.6)**	**51 (15.0)**
Unknown	527 (12.4)	**103 (11.0)**	**357 (12.0)**	**67 (19.7)**

a Boldface indicates *P* value <.05.

b Percentages do not total 100% due to rounding.

### 3.2. Cancer screening participation by occupational class

**Figure 1:**
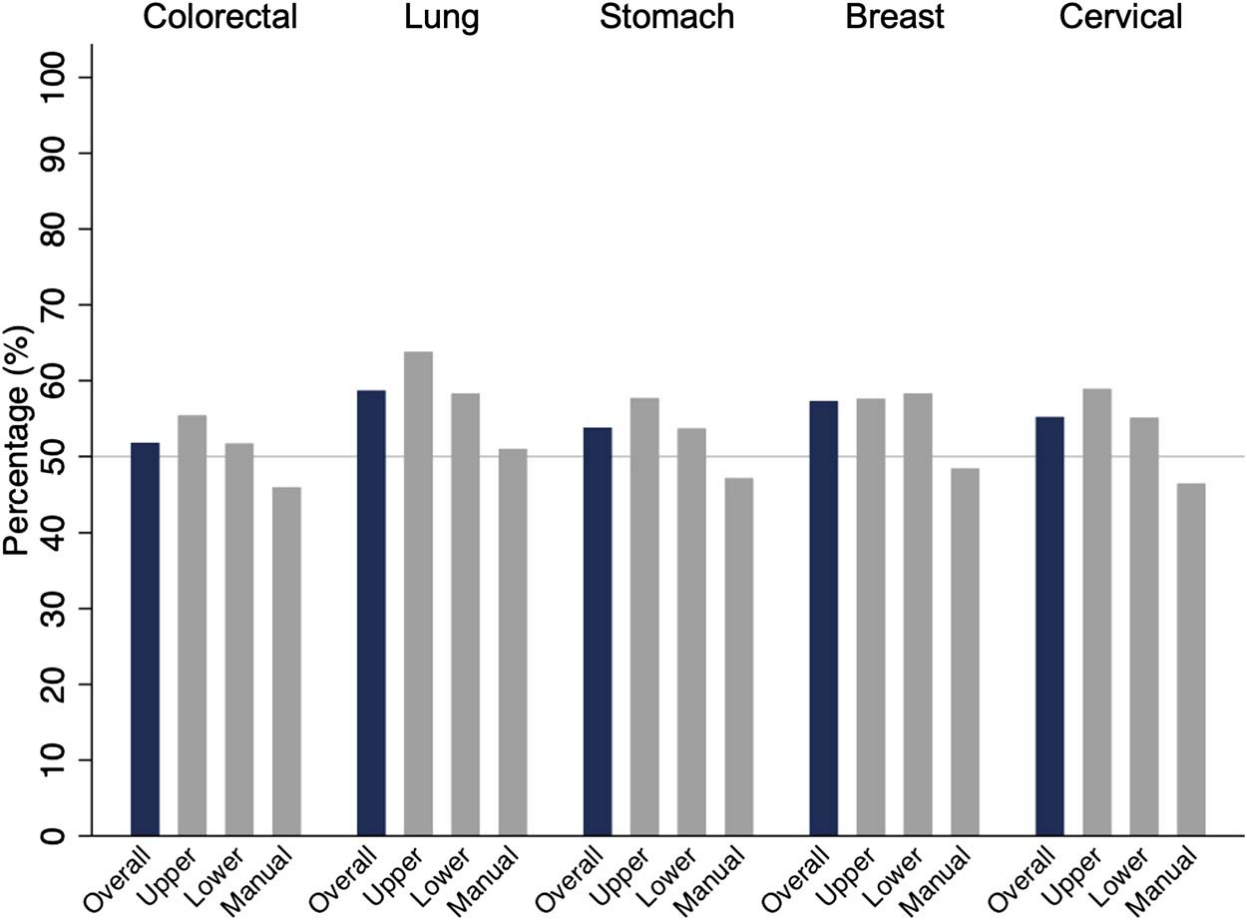
Cancer screening participation rates for each cancer site. The overall screening participation rates were 51.8% (3648/7038) for colorectal cancer, 58.7% (4130/7038) for lung cancer, 53.8% (3783/7038) for stomach cancer, 57.3% (1679/2929) for breast cancer, and 55.2% (2349/4252) for cervical cancer. Manual workers had lower participation rates for all cancer sites compared with other occupational classes: 46.0% (493/1071) for colorectal cancer, 51.0% (546/1071) for lung cancer, 47.2% (505/1071) for stomach cancer, 48.4% (124/256) for breast cancer, and 46.5% (158/340) for cervical cancer. In contrast, participation rates among upper nonmanual workers were 55.4% (1039/1874), 63.8% (1196/1874), 57.7% (1082/1874), 57.6% (338/587), and 58.9% (552/937), respectively, whereas those among lower nonmanual workers were 51.7% (2116/4093), 58.3% (2388/4093), 53.7% (2196/4093), 58.3% (1217/2086), and 55.1% (1639/2975), respectively. All *P* values for the chi-square test were <.05.

Overall, the screening participation rates for colorectal, lung, stomach, breast, and cervical cancers were 51.8%, 58.7%, 53.8%, 57.3%, and 55.2%, respectively (Tables 1, 2, and3; Figure 1). Across all cancer sites, manual workers had lower screening participation rates than other occupational classes, with rates of 46.0% for colorectal cancer, 51.0% for lung cancer, 47.2% for stomach cancer, 48.4% for breast cancer, and 46.5% for cervical cancer (Tables 1, 2, and3; Figure 1).

### 3.3. Nonparticipation in cancer screening (Poisson regression analysis)

Except for breast cancer (PR = 1.03; 95% CI, 0.87-1.20), manual workers had higher PRs for nonparticipation in screening across multiple cancer sites: colorectal (PR = 1.12; 95% CI, 1.04-1.22), lung (PR = 1.22; 95% CI, 1.12-1.34), stomach (PR = 1.14; 95% CI, 1.05-1.23), and cervical cancer (PR = 1.16; 95% CI, 1.02-1.33) (Model 2, Table 4).

**Table 4:** Results of Poisson regression analyses for nonparticipation in cancer screening (vs upper nonmanual workers).^a^

	**Prevalence ratio (95% CI)**		
	**Crude**	**Model 1** ^**b**^	**Model 2** ^**c**^
Colorectal (*n* = 7038)			
Lower nonmanual	**1.08 (1.02-1.15)**	**1.06 (1.00-1.13)**	1.06 (0.998-1.13)
Manual	**1.21 (1.12-1.31)**	**1.15 (1.06-1.24)**	**1.12 (1.04-1.22)**
Lung (*n* = 7038)			
Lower nonmanual	**1.15 (1.07-1.24)**	**1.13 (1.05-1.21)**	**1.12 (1.04-1.20)**
Manual	**1.35 (1.24-1.48)**	**1.25 (1.15-1.37)**	**1.22 (1.12-1.34)**
Stomach (*n* = 7038)			
Lower nonmanual	**1.10 (1.03-1.17)**	1.02 (0.96-1.09)	1.01 (0.95-1.08)
Manual	**1.25 (1.16-1.35)**	**1.16 (1.07-1.26)**	**1.14 (1.05-1.23)**
Breast (*n* = 2929)			
Lower nonmanual	0.98 (0.88-1.09)	0.91 (0.81-1.01)	0.90 (0.81-1.01)
Manual	**1.22 (1.04-1.41)**	1.06 (0.90-1.24)	1.03 (0.87-1.20)
Cervical (*n* = 4252)			
Lower nonmanual	**1.09 (1.00-1.19)**	1.04 (0.96-1.14)	1.05 (0.96-1.14)
Manual	**1.30 (1.15-1.48)**	**1.18 (1.04-1.35)**	**1.16 (1.02-1.33)**

a Boldface indicates *P* value <.05.

b Model 1: adjusted for age, sex, educational attainment, and household income.

c Model 2: adjusted for workplace scale.

### 3.4. Sensitivity analysis by age

**Figure 2:**
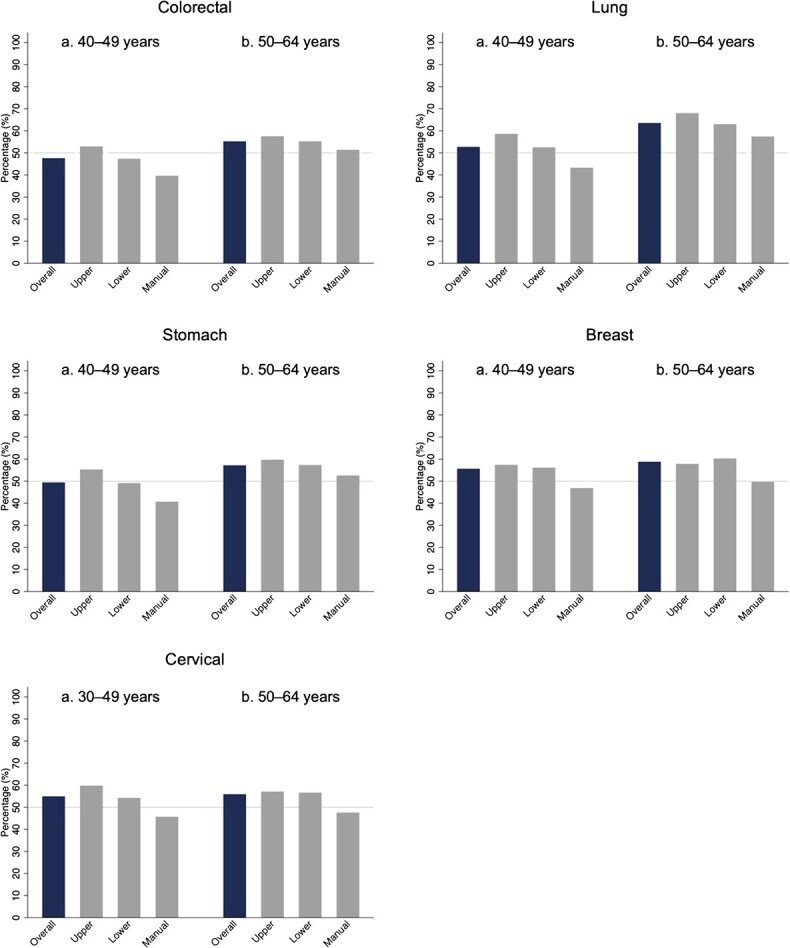
Age-stratified analyses of screening participation rates by cancer site. A, Among workers aged 40-49 years, the overall participation rates for colorectal, lung, stomach, and breast cancer screening were 47.6% (1490/3132), 52.7% (1650/3132), 49.4% (1548/3132), and 55.6% (758/1363), respectively. Participation rates for colorectal, lung, and stomach cancer were lowest among manual workers (39.6% [193/487], 43.3% [211/487], and 40.7% [198/487]), followed by lower nonmanual workers (47.3% [858/1815], 52.5% [953/1815], and 49.1% [891/1815]), and highest among upper nonmanual workers (52.9% [439/830], 58.6% [486/830], and 55.3% [459/830]), with significant differences observed across occupational classes. The participation rates for breast cancer screening were 57.4% (156/272) for upper nonmanual workers, 56.1% (549/978) for lower nonmanual workers, and 46.9% (53/113) for manual workers. The overall participation rate in cervical cancer screening was 54.9% (1474/2686) among workers aged 30-49 years. The participation rates were 59.8% (372/622) for upper nonmanual workers, 54.2% (1012/1867) for lower nonmanual workers, and 45.7% (90/197) for manual workers, with significant differences observed between the groups. B, Among workers aged 50-64 years, the overall participation rates for colorectal, lung, stomach, breast, and cervical cancer screening were 55.2% (2158/3906), 63.5% (2480/3906), 57.2% (2235/3906), 58.8% (921/1566), and 55.9% (875/1566), respectively. The participation rates of manual workers in colorectal, lung, stomach, breast, and cervical cancer screenings were 51.4% (300/584), 57.4% (335/584), 52.6% (307/584), 49.7% (71/143), and 47.6% (68/143), respectively. In comparison, participation rates among upper nonmanual workers were 57.5% (600/1044), 68.0% (710/1044), 59.7% (623/1044), 57.8% (182/315), and 57.1% (180/315), whereas those among lower nonmanual workers were 55.2% (1258/2278), 63.0% (1435/2278), 57.3% (1305/2278), 60.3% (668/1108), and 56.6% (627/1108), respectively. Significant differences were observed in lung, stomach, and breast cancer screening across occupational classes.

Sensitivity analyses revealed similar patterns, with more pronounced disparities among workers aged 40-49 years for colorectal, lung, and stomach cancer screening, and among those aged 30-49 years for cervical cancer screening (Figure 2 and Figure S1).

### 3.5. Subgroup analysis by sex

For colorectal, lung, and stomach cancer screening stratified by sex, participation rates were lower among both male (46.7%, 51.3%, and 49.3%, respectively) and female manual workers (43.8%, 50.0%, and 40.2%, respectively) compared with nonmanual workers (Figure S2). Additionally, the association between manual occupations and nonparticipation in screening was more pronounced among male workers.

## 4. Discussion

Using data from a nationwide cross-sectional internet survey in Japan, we investigated cancer screening participation rates among active workers across different occupational classes for cancers with recommended screenings. Our findings revealed that manual workers consistently had lower screening participation rates across all cancer sites. Furthermore, being in a manual occupation was significantly associated with nonparticipation in colorectal, lung, stomach, and cervical cancer screenings, with prevalence being 12%, 22%, 14%, and 16% higher, respectively, compared with upper nonmanual workers. These findings underscore the urgent need to enhance cancer screening participation among manual workers to mitigate occupational disparities in cancer prevention and outcomes.

Early cancer detection in the workplace is essential for improving health outcomes among workers. However, evidence on the relationship between occupational class and cancer screening participation remains limited.^15-17^ Previous research has shown that manual workers, particularly those in construction and production industries, have lower participation rates in colorectal, breast, and cervical cancer screenings.^16^ Manual workers often face barriers to accessing cancer screening and preventive healthcare compared with other occupational classes, due to long working hours, irregular shifts, and physically demanding labor. Additionally, blue-collar workers often experience high levels of work-related stress^18^ and depression,^19^ which may reduce their willingness to seek medical care^20^ and participate in cancer screening. Perceptions of health may also influence screening participation, as individuals with poor self-rated health may be less likely to engage in proactive health behaviors.^21^ Addressing these barriers requires a comprehensive understanding of the work environment and occupational characteristics of manual workers, along with the development of targeted interventions to facilitate their access to cancer screening.

In comparing upper nonmanual workers with lower nonmanual workers, it was observed that the latter group exhibited a lower likelihood of participation in cancer screenings. This disparity may be attributed to the more flexible working hours, increased opportunities for engagement in screenings and health promotion activities, and heightened health awareness among upper nonmanual workers. Conversely, lower nonmanual workers may miss opportunities for screenings due to differences in working conditions, such as the ability to take leave, when compared with their upper nonmanual counterparts.

When comparing lower nonmanual workers with manual workers, these barriers to screening participation are likely even more pronounced among manual workers. However, the participation rate in breast cancer screening was higher among lower nonmanual workers than among upper nonmanual workers. Among female participants in breast cancer screenings it is conceivable that the relatively large workplace size of lower nonmanual workers facilitates active encouragement and implementation of workplace screenings.^22^ Additionally, health literacy,^23^ awareness activities, and workplace culture^24^ related to breast cancer screenings may influence this trend among lower nonmanual workers.

In contrast, the participation rate for lung cancer screening was lower among lower nonmanual workers than among upper nonmanual workers. People who use tobacco products may have a lower likelihood of participating in lung cancer screening.^25^ Although the exact smoking rate remains uncertain, lower nonmanual workers may exhibit a higher prevalence of smoking. This may contribute to diminished awareness of lung cancer risk and, subsequently, lower screening rates.

In terms of age, the association between manual occupations and nonparticipation in cancer screening was more pronounced among individuals aged ≤49 years, highlighting the importance of promoting cancer screening in younger working populations. In contrast, this association was less consistent among older age groups and varied by cancer site, suggesting the need for further investigation into the age-dependent nature of this relationship. Workers’ health awareness and behaviors may vary with age, influenced by social networks at home and in the workplace.^26^ Subgroup analysis revealed that the association between manual occupations and nonparticipation in colorectal, lung, and stomach cancer screening was particularly strong among male workers. Understanding sex-based differences in cancer screening participation is crucial for designing effective interventions to improve screening uptake.^27^ Mobile screening units^28^ and workplace- or community-based group screening programs may enhance women’s participation in breast cancer screening. In contrast, cervical cancer screening is primarily conducted in clinics or specialized facilities. Shift work and irregular working hours can contribute to occupational disparities in access and participation.

This study has some limitations. First, generalizability is limited because job transitions and long-term occupational histories could not be assessed. Since occupational classification was based solely on the participants’ current occupations at the time of the survey, it may not have fully captured past occupational experiences or career trajectories. However, all participants had current occupational information, allowing for classification based on predefined occupational categories. Second, because the study population was limited to individuals with the cognitive ability and skills to complete an internet survey, a selection bias may have been introduced, potentially favoring those with higher education or health awareness. Therefore, our findings may overestimate overall cancer screening participation rates, and the actual participation among manual workers may be even lower. Additionally, information bias may have led to either an underestimation or overestimation of screening participation rates because of substantial missing data on breast and cervical cancer screening. Third, due to the cross-sectional study design, causal relationships could not be inferred. Further research is needed to elucidate the mechanisms linking occupational class and participation in cancer screening.

Despite these limitations, to the best of our knowledge, this nationwide study is the first to establish an association between occupational class and cancer screening participation in Japan. Our findings underscore the critical need to enhance cancer screening among manual workers to address disparities in preventive healthcare. Given the poor cancer survival outcomes observed in this occupational group, improving screening participation is an urgent public health priority. The implementation of targeted interventions to enhance early cancer detection and timely treatment can significantly improve health outcomes. Efforts to improve health literacy and workplace environments are essential for raising awareness of preventive health behaviors among manual workers and fostering equitable healthcare access across occupational classes.

In conclusion, manual occupations were associated with higher nonparticipation in colorectal, lung, stomach, and cervical cancer screenings. Targeted efforts should focus on increasing awareness of cancer screening among manual workers and improving workplace accessibility to screening services, particularly among middle-aged workers. Future longitudinal studies or intervention trials should assess whether tailored workplace screening programs reduce disparities among manual workers.

## Supplementary Material

Web_Material_uiaf046

## Data Availability

Data supporting the findings of this study are restricted due to personal identification and privacy concerns. The research data can be obtained from the corresponding author upon reasonable request.
