# Supplementary material for: Occupational disparities in common cancer screening participation among workers: a nationwide cross-sectional study in Japan
Source: J Occup Health. 2025 Sep 8;67(1):uiaf046. doi: 10.1093/joccuh/uiaf046 (PMC12445672; doi:10.1093/joccuh/uiaf046)
Supplement: Web_Material_uiaf046 [file web_material_uiaf046.zip › Supplementary_material.docx]

**Supplementary material**

**Figure S1. Association between occupational class and non-participation in cancer screening stratified by age**

The prevalence ratio (circles) and 95% confidence intervals (bars) of non-participation in cancer screenings compared to upper non-manual workers were estimated using Poisson regression with robust variance. We adjusted for sex, age, educational level, household income, and workplace size.

The analytical samples were as follows:

A. Colorectal, lung, stomach, and breast cancer screenings

1. n = 3,132 (830 upper non-manual workers, 1,815 lower non-manual workers, and 487 manual workers) underwent colorectal, lung, and stomach cancer screening (aged 40–49 years).

2. n = 1,363 (272 upper non-manual workers, 978 lower non-manual workers, and 113 manual workers) underwent breast cancer screening (aged 40–49 years).

3. n = 3,906 (1,044 upper non-manual workers, 2,278 lower non-manual workers, and 584 manual workers) underwent colorectal, lung, and stomach cancer screening (aged 50–64 years).

4. n = 1,566 (315 upper non-manual workers, 1,108 lower non-manual workers, and 143 manual workers) underwent breast cancer screening (aged 50–64 years)

B. Cervical cancer screening

(5) n = 2,686 (622 upper non-manual workers, 1,867 lower non-manual workers, and 197 manual workers) (aged 30–49 years)

(6) n = 1,566 (315 upper non-manual workers, 1,108 lower non-manual workers, and 143 manual workers) (aged 50–64 years)

**Figure S2. Participation rates for colorectal, lung, and stomach cancer screenings by occupational class and sex**

Overall, the participation rates for colorectal, lung, and stomach cancer screening among men and women were 52.0% (2,138/4,109) and 51.6% (1,510/2,929); 58.6% (2,409/4,109) and 58.8% (1,721/2,929); and 57.6% (2,366/4,109) and 48.4% (1,417/2,929), respectively. The participation rates were lower among male and female manual workers with 46.7% (381/815) and 43.8% (112/256) for colorectal cancer, 51.3% (418/815) and 50.0% (128/256) for lung cancer, and 49.3% (402/815) and 40.2% (103/256) for stomach cancer, respectively. Among the upper non-manual workers, the participation rates were 55.9% (720/1,287) and 54.3% (319/587) for colorectal cancer, 62.8% (808/1,287) and 66.1% (388/587) for lung cancer, and 61.8% (795/1,287) and 48.9% (287/587) for stomach cancer in men and women, respectively. Among lower non-manual workers, participation rates were 51.7% (1,037/2,007) for colorectal cancer, 58.9% (1,183/2,007) for lung cancer, and 58.2% (1,169/2,007) for stomach cancer in men. In women, participation rates were 51.7% (1,079/2,086) for colorectal cancer, 57.8% (1,205/2,086) for lung cancer, and 49.2% (1,027/2,086) for stomach cancer. All p-values for the chi-square test were <0.05.

**Figure S3. Prevalence ratios and 95% confidence intervals for non-participation in colorectal, lung, and stomach cancer screenings (vs. upper non-manual workers) by sex**

Prevalence ratios (PRs [circles]) and 95% confidence intervals (95% CIs [bars]) for non-participation in cancer screening, compared to upper non-manual workers, were estimated using Poisson regression with robust variance. We adjusted for age, educational attainment, household income, and workplace size. Among men, manual workers had significantly higher PRs for non-participation in colorectal (PR=1.13, 95% CI: 1.03–1.24), lung (PR=1.20, 95% CI: 1.08–1.34), and stomach cancer screenings (PR=1.17, 95% CI: 1.06–1.30). The number of participants for colorectal, lung, and stomach cancer screenings among men was 1,287 in upper non-manual workers, 2,007 in lower non-manual workers, and 815 in manual workers; among women, the number of participants was 587, 2,086, and 256, respectively.
