# Supplementary figures and images for: Occupational disparities in common cancer screening participation among workers: a nationwide cross-sectional study in Japan
Source: J Occup Health. 2025 Sep 8;67(1):uiaf046. doi: 10.1093/joccuh/uiaf046 (PMC12445672; doi:10.1093/joccuh/uiaf046)

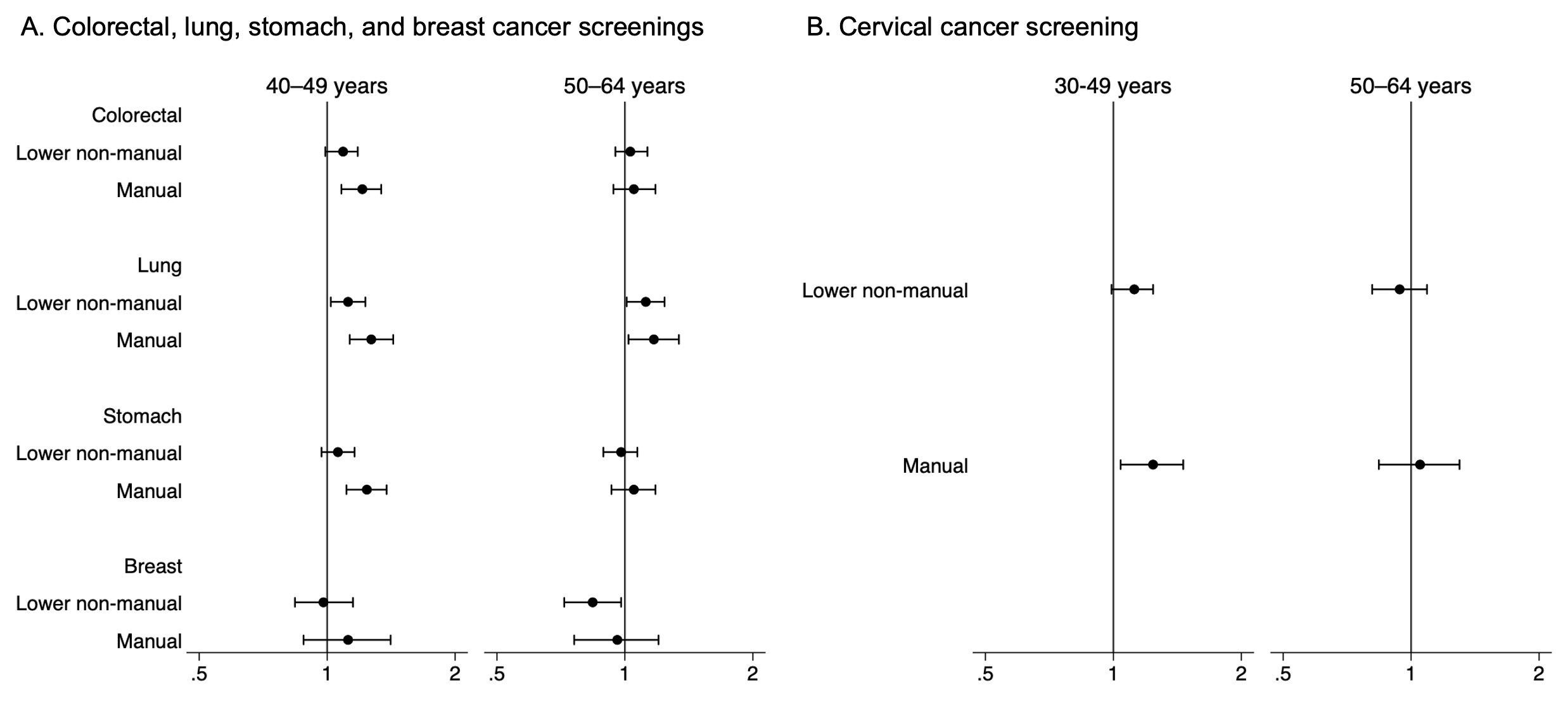

Supplement: Web_Material_uiaf046 [file web_material_uiaf046.zip › Figure_s1.jpg]

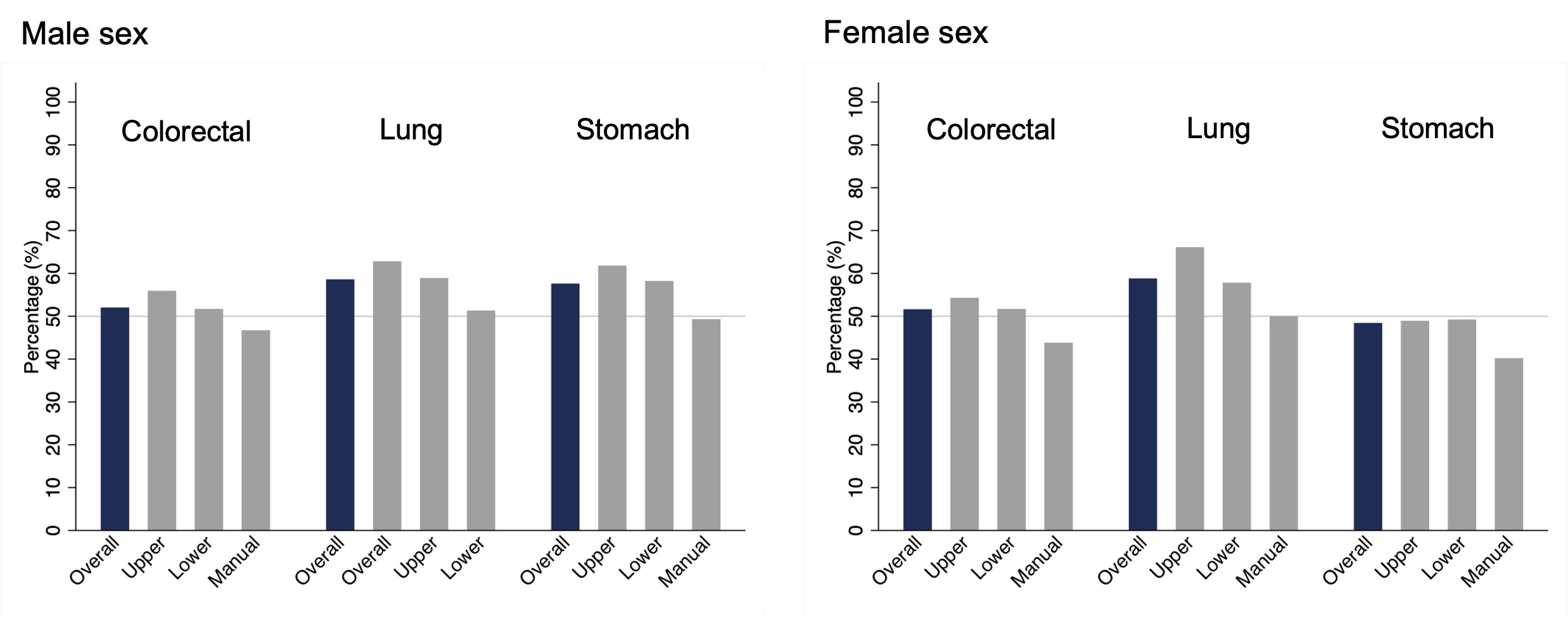

Supplement: Web_Material_uiaf046 [file web_material_uiaf046.zip › Figure_s2.jpg]

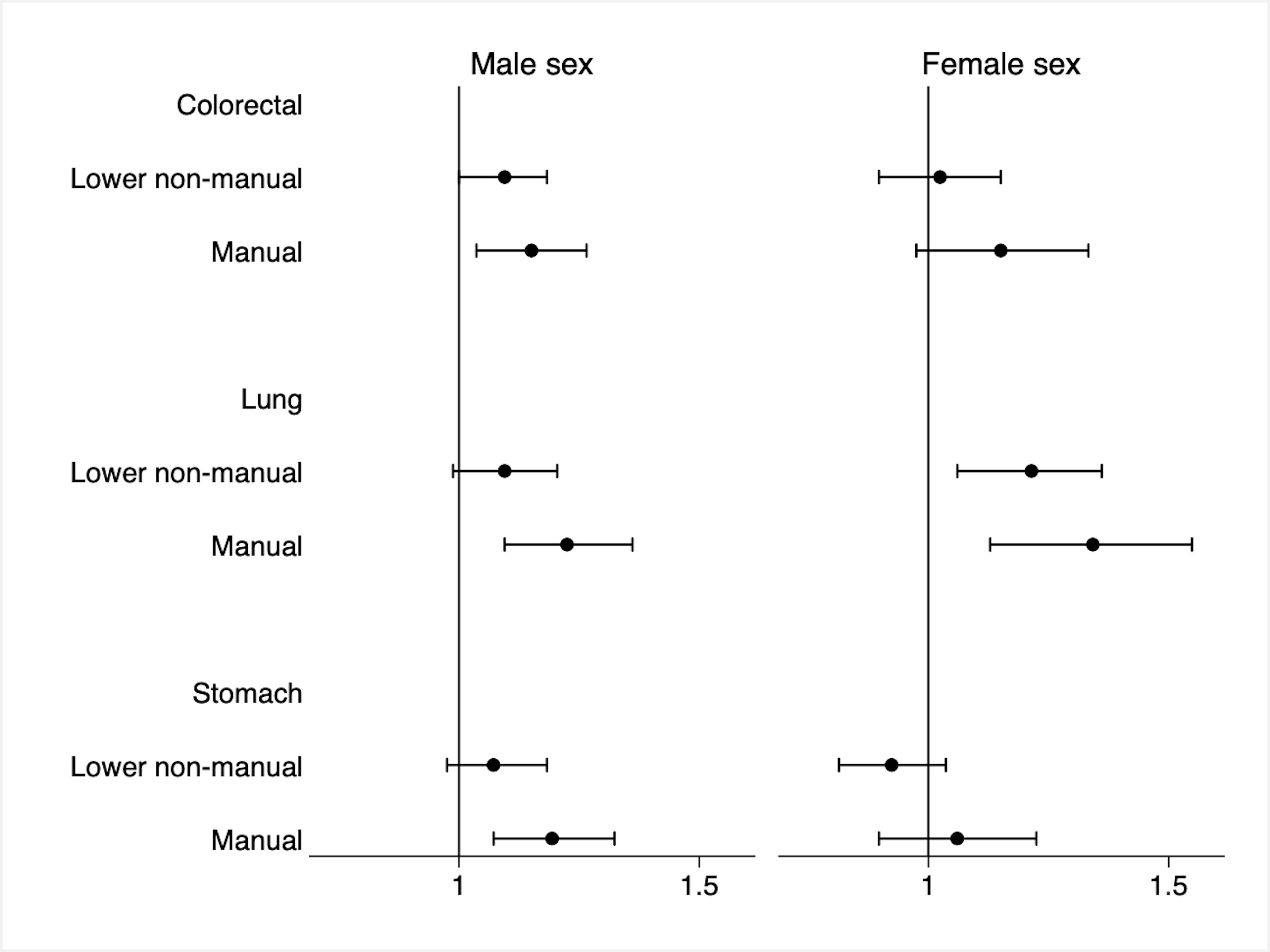

Supplement: Web_Material_uiaf046 [file web_material_uiaf046.zip › Figure_s3.jpg]
